# Safe administration of etoposide phosphate after hypersensitivity reaction to intravenous etoposide

**DOI:** 10.1038/sj.bjc.6600003

**Published:** 2002-01-07

**Authors:** J Siderov, P Prasad, R De Boer, J Desai

**Affiliations:** Pharmacy Department, Austin & Repatriation Medical Centre, Studley Road, Heidelberg, Australia 3084; Cancer Services, Austin & Repatriation Medical Centre, Studley Road, Heidelberg, Australia 3084

**Keywords:** etoposide, etoposide phosphate, hypersensitivity, adverse drug reaction

## Abstract

Etoposide is commonly used in a variety of malignancies. A well known but rare toxicity are hypersensitivity reactions, usually manifested by chest discomfort, dyspnoea, bronchospasm and hypotension. We report the details of a patient who developed hypersensitivity reactions to intravenous etoposide, but subsequently tolerated the administration of intravenous etoposide phosphate with no sequalae.

*British Journal of Cancer* (2002) **86**, 12–13. DOI: 10.1038/sj/bjc/6600003 www.bjcancer.com

© 2002 The Cancer Research Campaign

## 

The podophyllotoxin etoposide has been used clinically for over 30 years. It is active in the treatment of a variety of malignant conditions and can be administered in either an i.v. or in an oral form. Intravenous etoposide is generally well tolerated. A well known but rare toxicity is a type I hypersensitivity reaction, manifested by dyspnoea, chest discomfort, hypotension, bronchospasm and/or skin flushing (Weiss, 1996; O'Brien and Souberbielle, 1992). It remains unclear whether this reaction is due to either the active drug or the solvent (Weiss, 1996).

We report here the details of a patient who, despite experiencing a hypersensitivity reaction to intravenous etoposide, tolerated the subsequent administration of intravenous etoposide phosphate without any allergic sequalae. A test dose of etoposide phosphate was not administered, and premedication, which was administered initially, was discontinued. This case highlights three important aspects in patients experiencing hypersensitivity reaction to etoposide: (i) etoposide phosphate can be considered as an appropriate alternative; (ii) premedication may not be required; and (iii) the hypersensitivity reaction is more likely due to the solvent.

## CASE DESCRIPTION

A 19-year-old male with a newly diagnosed primary mediastinal non-seminomatous germ cell tumour was admitted for treatment with the standard BEP regimen. This consisted of weekly i.v. bleomycin 30 000 iu and 3-weekly cycles of i.v. etoposide 100 mg m^−2^ day^−1^ for 5 days and cisplatin 20 mg m^−2^ day^−1^ for 5 days (Williams *et al*, 1987). The patient had no known allergies. Following premedication with i.v. tropisetron (5 mg) and dexamethasone (8 mg) administration of etoposide was commenced (200 mg in 500 ml of sodium chloride 0.9%, over 60 min). Within minutes of commencement of the first dose of etoposide the patient complained of generalized discomfort and shortness of breath. He was found to be hypotensive (BP=95/60), tachycardic (PR=110) and had an oxygen saturation of 80% on room air. The infusion was immediately ceased. Treatment was begun with i.v. hydration, with bolus doses of hydrocortisone (100 mg) and promethazine (50 mg). Oxygen (6 l min^−1^) and nebulized salbutamol were also given. The patient improved rapidly with complete resolution of his symptoms, and was comfortable within 1 h. No bleomycin or cisplatin was administered.

Germ cell tumours of the mediastinum are potentially curable, and etoposide is considered to be a critical component of an effective treatment schedule. Thus, it was felt that in this case continued use of etoposide was warranted. In light of this decision, cycle 1 of chemotherapy was recommenced a few days later but with etoposide replaced by the equivalent dose of etoposide phosphate. The patient was premedicated with hydrocortisone (100 mg) and promethazine (25 mg). No reaction occurred with the administration of the etoposide phosphate and the cisplatin and bleomycin were also administered without problem. The same procedure was followed the next day (day 2) with the same specific pre-medication followed by etoposide phosphate and then cisplatin. Again, there was no evidence of a HSR. As the patient experienced drowsiness with promethazine, and in view of the fact that there had been no further evidence of a HSR, the last three doses of etoposide phosphate in cycle 1 were given without specific anti-HSR premedication, although oral dexamethasone at a dose of 4 mg twice daily was given as anti-emetic prophylaxis. There was no evidence of a HSR on any of these days. Subsequent cycles of BEP have been administered using standard anti-emetic pre-medication but with no histamine antagonists, and there have been no complications.

## DISCUSSION

Hypersensitivity reactions occur as an adverse effect of cancer chemotherapy in up to 40% of patients (Weiss, 1996; O'Brien and Souberbielle, 1992). Etoposide has been reported to cause a hypersensitivity reaction in 1–3% of patients (Weiss, 1996), but one study observed hypersensitivity reactions in 33% of children treated for acute leukaemia (Kellie *et al*, 1991). In most patients the reactions occur within the first 5 to 10 min of infusion and complete recovery is usual once the infusion is discontinued. However, hypersensitivity reactions have been reported up to several hours after administration (Weiss, 1996). There are no known risk factors for developing a HSR to etoposide, and in few of the reported cases do the patients have a history of drug allergy (Kasperek and Black, 1992; Bernstein and Troner, 1999; Athanassiou *et al*, 1988; De Souza *et al*, 1994; Tester *et al*, 1990; Hoetelman *et al*, 1996; Siderov and Zalcberg, 1994; Tucci and Pirtoli, 1985; Donegan, 1989; Eschalier *et al*, 1988; Schacter, 1996).

The mechanism underling hypersensitivity reactions to epipodophyllotoxins have not been fully elucidated. Typically, type I HSR occur, although type II reactions have also been reported (Weiss, 1996; O'Brien and Souberbielle, 1992; Kasperek and Black, 1992). Bernstein and Troner (1999) have attributed etoposide HSRs to the concentration of the drug and the rate of infusion. However, other authors have reported HRSs in patients receiving a wide range of etoposide concentrations, weakening this hypothesis (Kasperek and Black, 1992; Bernstein and Troner, 1999; Athanassiou *et al*, 1988; De Souza *et al*, 1994; Tester *et al*, 1990; Hoetelman *et al*, 1996; Siderov and Zalcberg, 1994; Tucci and Pirtoli, 1985; Donegan, 1989; Eschalier *et al*, 1988; Schacter, 1996). Another suggested hypothesis is that the vehicle used to dissolve the etoposide (benzyl alcohol and polysorbate (tween) 80) is responsible for the HSR (Weiss, 1996). In animal models, polysorbate has been shown to induce histamine release and cause hypersensitivity reactions (Eschalier *et al*, 1988). The fact that there have been no reports of hypersensitivity reactions caused by oral etoposide, a formulation containing citric acid, glycerin and polyethylene glycol, but no polysorbate 80, provides additional support for this hypothesis (Weiss, 1996). Etoposide phosphate, a water soluble prodrug of etoposide, was designed to obviate problems associated with etoposide (Schacter, 1996). Formulations of etoposide phosphate do not contain polysorbate 80 and there have been no case reports in the literature of HSR to etoposide phosphate. Therefore the substitution of etoposide phosphate in patients who experience a HSR to etoposide rather than rechallenge would seem appropriate. Another possible alternative is the use of oral etoposide (Siderov and Zalcberg, 1994).

Our patient was retreated using etoposide phosphate at an equivalent dose. The patient was premedicated with hydrocortisone (100 mg) and promethazine (25 mg) and no test dose of etoposide phosphate was administered. After 2 days of therapy with etoposide phosphate, the specific HSR premedication was discontinued. However, the patient continued to have oral dexamethasone (4 mg twice daily) as anti-emetic prophylaxis. No HSR occurred at any stage of cycle 1, and subsequent cycles of BEP have been administered with no complications.

This case highlights that patients experiencing HSR to etoposide can be successfully treated with etoposide phosphate without adverse sequalae. Premedication appears prudent as initial therapy, but can be successfully weaned. This case supports the hypothesis that polysorbate 80 is a likely causative factor in HSRs to etoposide. The only other published report of a successful substitution with etoposide phosphate in a patient who experienced a HSR to etoposide did not conclude whether the premedication schedule used (which included both H_1_ and H_2_ antihistamine agents) or the use of etoposide phosphate was the factor preventing the HSR from reoccurring (Bernstein and Troner, 1999).

Hypersensitivity reactions to etoposide are uncommon, but can be life threatening. The subsequent management of patients experiencing a HSR to etoposide is usually the omission of etoposide from the chemotherapy regimen (Athanassiou *et al*, 1988; De Souza *et al*, 1994; Tucci and Pirtoli, 1985; Donegan, 1989). However, since etoposide is considered a critical component of therapy, its omission in a young patient with a potentially curable malignancy was thought inappropriate. Therefore, in similar situations where subsequent doses of etoposide are deemed necessary, replacement with etoposide phosphate is a reasonable and viable alternative.
